# The impact of parental absence on the mental health of middle school students in rural areas of Western China

**DOI:** 10.3389/fpubh.2025.1439799

**Published:** 2025-03-04

**Authors:** Xiaohong Ren, Cen Lin, Lu Pan, Qiuyue Fan, Dapeng Wu, JinLong He, Ping He, Jiaming Luo

**Affiliations:** ^1^Department of Hematology, The Affiliated Hospital of North Sichuan Medical College, Nanchong, China; ^2^School of Psychiatry, North Sichuan Medical College, Nanchong, China

**Keywords:** divorced family, migration, depression, anxiety, mental health, middle school students, China

## Abstract

**Background:**

Extensive research has established the association between parental absence and adolescent psychological well-being, particularly in the Chinese context. However, studies specifically examining the dual impact of parental separation and migration on psychological outcomes among adolescents in Western China remain relatively limited.

**Aim:**

This study aims to systematically examine the association between various parental absence situations and mental health outcomes in early adolescence, with the objective of informing targeted interventions and policy formulations to optimize psychosocial support systems for vulnerable youth population.

**Methods:**

The Wilcoxon rank-sum test was employed to analyze continuous and ordinal variables that exhibited non-normal distributions. To investigate the associations between various patterns of parental absence and psychological outcomes (depression, anxiety, and stress) among middle school students, binary logistic regression analysis was performed, while the model’s goodness-of-fit was evaluated by using the Hosmer-Lemeshow test, with a *p* > 0.05 indicating satisfactory model fit.

**Results:**

This cross-sectional study investigated mental health outcomes among a cohort of 8,606 middle school students, revealing notable prevalence rates of depressive symptoms (6.7%), anxiety (6.1%), and stress-related symptoms (8.1%). Multivariate analysis demonstrated that different forms of parental absence exerted substantial effects on mental health severity, with statistically significant associations for depression, anxiety, and stress (all *p* < 0.001). The results revealed that various forms of parental absence had a significant impact on depression, anxiety, and stress. Specifically, the combined impact of divorce and left-behind children (DLC) creates a synergistic effect, resulting in psychological risks (OR = 1.623–1.725, all *p* < 0.001), that are significantly higher than those associated with either factor individually (LBC/DC). Further analysis identified additional risk factors, including senior high school (OR = 1.486, *p* < 0.001), boarding school (OR = 1.155, *p* = 0.037), and girls (anxiety OR = 1.213, *p* < 0.001), all showing significant associations with adverse mental health outcomes.

**Conclusion:**

Our study underscores significant mental health risks associated with diverse patterns of parental absence among adolescents in the Sichuan region. By fostering stronger parent–child bonds and providing targeted emotional support, it may be possible to mitigate the adverse psychological effects of parental absence and help adolescents better navigate these mental health challenges.

## Introduction

UNICEF’s latest mental health research reveals that nearly 25% of adolescents suffer from mild to severe depression. Over 30 million children and adolescents in China are reported to be struggling with emotional or behavioral challenges (1). Amidst significant economic, cultural, and social changes, adolescents are encountering a variety of health issues, particularly related to mental well-being. Studies indicate that adverse childhood experiences are the leading risk factors for the development of mental health conditions (2).

Research has consistently demonstrated that parental marital status significantly elevates the risk of depression among children. Specifically, children from separated or divorced families are 2 to 3 times more likely to experience depression compared to those from intact families (3). Among adolescents, parental separation is strongly correlated with increased anxiety. Empirical studies reveal that the prevalence of depression and anxiety in children from divorced families is 1.29 times and 1.12 times higher respectively, than in children from intact families (4). The separation from parents during childhood can have both immediate and enduring effects, not only contributing to depression and anxiety but also elevating the risk of behavioral problems (5), learning difficulties (6), and substance abuse (7).

Similar to other developing nations, China has undergone rapid economic growth and urbanization in recent decades, leading to substantial shifts in social structures and family dynamics. Notably, rural areas in western China, characterized by significant economic disparities, have witnessed frequent marital conflicts arising from daily life and financial pressures. These conflicts are often exacerbated by challenging living conditions. Furthermore, the increasing trend of labor migration has intensified feelings of estrangement and conflict between spouses, which are identified as key factors driving the rising divorce rate (8, 9). Consequently, the number of adolescents residing in divorced families has shown a steady increase.

With the rapid acceleration of urbanization and industrialization in China, a significant migration of individuals from rural to urban areas has occurred. Many parents, seeking employment opportunities, relocate to urban centers, leaving their children behind in rural areas. This phenomenon has led to the widespread prevalence of left-behind children (LBC), an issue deeply intertwined with the broader processes of globalization and urbanization. The distribution of LBC varies significantly across regions, with an estimated 68.7 million children under the age of 18 being left behind in their hometowns by migrating parents (10). Sichuan Province, situated in western China, is one of the most densely populated provinces in the country and exhibits a particularly high proportion of LBC in its rural areas. This region accounts for 11.34% of the total LBC population nationwide (11).

Prolonged separation from parents can engender feelings of helplessness and loneliness in children, as they are deprived of the essential parental guidance and support necessary for their cognitive, emotional, and moral development. The lack of emotional attention and care during critical developmental stages may result in distorted values and worldviews, as well as abnormal psychological and personality development. Consequently, these children often become one of the most marginalized and vulnerable groups within Chinese society (12). Empirical studies indicate that approximately 8 to 29% of LBC are at risk of developing mental health issues. Research consistently underscores that parental absence, extended periods of separation, and the duration of being left behind can significantly impair a child’s overall health, particularly their social and psychological well-being (13).

A comprehensive study utilizing data from the China Education Panel Survey (CEPS) has revealed significant disparities in health outcomes between LBC and their counterparts from intact rural families. The findings indicate that LBC exhibit substantially poorer physical and mental health status (14). Empirical evidence demonstrates that parental migration significantly increases the risk of mental health disorders among affected children (15), with a particularly pronounced vulnerability to depressive symptoms (16). Furthermore, the research establishes a strong correlation between parental migration and both internalizing and externalizing behavioral problems. Specifically, children experiencing parental migration show elevated levels of internalizing problems, including depression, anxiety, and psychological distress (17), as well as externalizing issues such as hyperactivity (18).

The rising prevalence of divorce and parental separation due to migration has generated significant societal concern regarding their compounded psychological impacts on children. When these two disruptive events co-occur, minors face complex psychosocial challenges as they simultaneously overcome the trauma of family dissolution and prolonged parental absence. This dual adversity may exacerbate psychological vulnerabilities, potentially manifesting as intensified feelings of worthlessness, social isolation, anxiety disorders, and persistent fear responses. Empirical evidence from Southeast Asia corroborates these concerns. A Vietnamese adolescent study revealed significantly elevated risks of stress disorders (28.3%), clinical anxiety (18.9%), and depressive symptoms (32.1%) among youth experiencing parental separation compared to intact family peers (19). Complementary longitudinal analysis by Mao (20) utilizing nationally representative datasets China Education Panel Survey (CEPS 2013–2014) established significant correlations between parental absence and both deteriorating mental health indicators and reduced academic engagement. Regional studies in China further delineate specific behavioral consequences. Wang’s Anhui cohort study (21) identified marked differences in divorced-family adolescents from divorced families exhibit more internalizing and externalizing problems, display lower levels of prosocial behavior, and are more prone to having suicidal thoughts and engaging in non-suicidal self-injury. Zhao’s comparative analysis across Zhejiang and Guizhou provinces (22) revealed differential psychosocial impacts, with current and former LBC experiencing 1.8-times greater divorce-related distress than non-migrant peers. Notably, social support mechanisms showed enhanced protective effects for these doubly disadvantaged groups, particularly through peer networks and community programs.

While extant research has extensively documented the psychological consequences of parental absence among Chinese adolescents, critical knowledge gaps persist regarding two underexplored dimensions: (1) the synergistic effects of marital dissolution and parental migration, and (2) region-specific manifestations in understudied western China. Our investigation addresses these dual limitations through a targeted examination of middle school students in China’s western regions, employing a novel four-category parental absence typology: non-divorced, non-left-behind children(NLBC), non-divorced with left-behind children (LBC), divorced and non-left-behind children(DC), divorced and left-behind children (DLC). This analytical framework enables systematic comparison of mental health across distinct family configurations, particularly elucidating how migration trajectories interact with marital status to compound or mitigate adolescent distress. By focusing on the socioeconomically diverse western provinces, where labor export patterns and cultural norms differ substantially from coastal regions, the study generates crucial evidence for developing context-sensitive mental health interventions.

## Methods

### Procedure

We contacted the principals of the 23 participating schools to request their cooperation and authorization for managing the questionnaires. Data for this study were collected during March and April 2020. The region comprises 20 townships. A stratified random sampling method was used, with schools as the sampling units. One school was selected from each of the 17 smaller townships, and two schools were selected from each of the three larger townships, resulting in 23 schools participating in the study. Surveyors then entered the questionnaires into the “Wenjuanxing” platform, a professional online survey tool (website),^1^ and generated QR codes. These QR codes were distributed to parents or guardians by the schools. Upon receiving informed consent from the parents or guardians, participants were able to scan the QR code. The informed consent form appeared on the first page, and participants could proceed with completing the questionnaire only after providing their consent.

**Figure 1:**
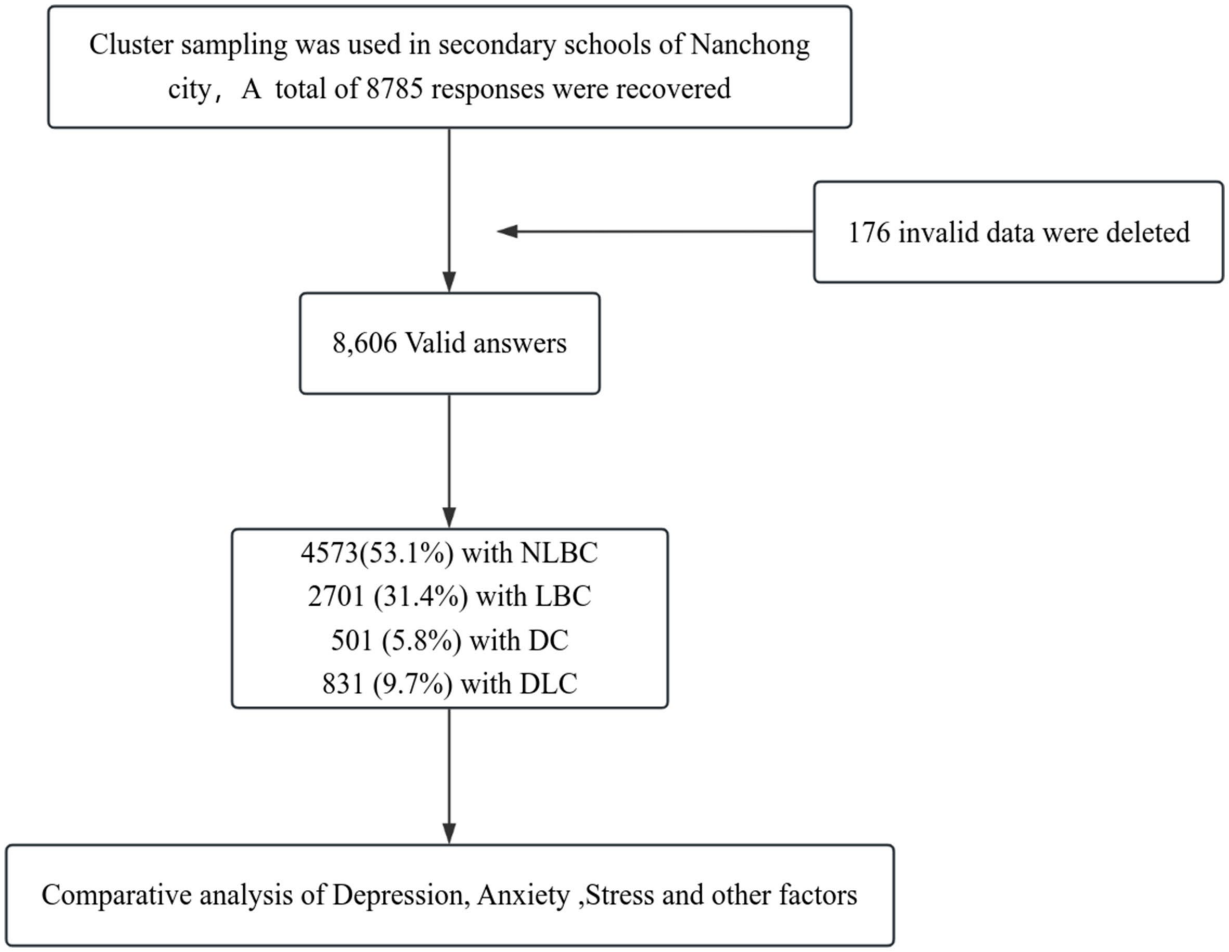
Flowchart of sample selection.

A total of 8,785 questionnaires were distributed to students from 23 high schools, yielding 8,606 valid responses, resulting in an effective response rate of 98.0%. A total of 176 individuals (2.0%) were excluded due to refusal of informed consent, missing data, obvious fabrication, or inconsistent responses. The inclusion criteria for adolescents with divorced parents were as follows: children under 18 years of age (ages 12 to 18) whose parents had been divorced or separated. This study was conducted in accordance with ethical principles and reviewed by the Ethics Committee of Nanchong Psychosomatic Hospital (Figure 1).

### Measures

The General Information Questionnaire is self-administered and gathers socio-demographic data from secondary school students, including age, gender, education level (junior high school or senior high school), and parental marital status. Children who have experienced parental separation or divorce and have not lived with both parents are categorized as having experienced parental divorce. The questionnaire also covers parental migration (for children under 18, where one or both parents work away from home and the child has been unable to live with both parents for 6 months or above), and boarding school (students who reside at school for at least 5 days a week during the academic year).

Inclusion and exclusion criteria: (1) Strict inclusion and exclusion criteria were applied to identify participants for the current study: (a) For LBC (Left-Behind Children due to Parental Migration), the parents were currently working away from home (non-divorced families); for NLBC (Non-Left-Behind Children), both parents lived in the household and neither had ever worked away from home (non-divorced families); (b) For DC (Divorced Children), children living in divorced families without migrant parents; for DLC (Divorced and Left-Behind Children), children living in divorced families with migrant parents. Exclusion criteria: (1) Children whose one or both parents are deceased; (2) Children living in stepfamilies. A total of 4,573 NLBC, 2,701 LBC, 501 DC, and 831 DLC were ultimately included.

### Depression anxiety stress scale-21

We used the validated Chinese version of the Depression Anxiety Stress Scale-21 (DASS-21) to assess psychological distress (23). This concise 21-item instrument contains three subscales (7 items each) measuring stress, anxiety, and depression, adapted from Lovibond’s original 42-item scale through factor loading optimization (24). Participants rated symptom frequency over the past week using a 4-point Likert scale (0–3). Following standard scoring protocols, subscale scores are doubled to achieve a 0–42 range per dimension, with higher scores indicating greater severity. Established clinical thresholds are: Stress (0–14 = normal, 15–18 = mild, 19–25 = moderate, ≥26 = severe; cutoff >17); Anxiety (0–7 = normal, 8–9 = mild, 10–14 = moderate, ≥15 = severe; cutoff >7); Depression (0–9 = normal, 10–13 = mild, 14–20 = moderate, ≥21 = severe; cutoff >9) (25, 26). The scale demonstrates strong psychometric properties in Chinese adolescents, with reported internal consistency (*α* = 0.82–0.97) (27, 28). In our study, Cronbach’s alpha values were 0.83 for Stress, 0.83 for Anxiety, and 0.86 for Depression, confirming its reliability for this population (23).

### Statistical analyses

The data were analyzed by using SPSS 26.0. The Kolmogorov–Smirnov test (K-S test) revealed that the data did not follow a normal distribution. Non-parametric tests were used for group comparisons to evaluate the medians. For non-normally distributed continuous and ordinal variables, the Wilcoxon rank-sum test was applied. Furthermore, binary logistic regression analysis was conducted to examine the association between different forms of parental absence and depression, anxiety, and stress in middle school students. Risk factors were identified, and the Hosmer-Lemeshow test was used to assess the model’s goodness of fit. *p <* 0.05 indicated a good fit of the model. The significance level was set at *α* = 0.05 (two-tailed).

## Results

### Socio-demographic data

The cohort consisted of 8,606 middle school students, with a median age of 14 years. Among them, 4,540 were girls (52.8%) and 4,066 were boys (47.2%). Of the total, 5,937 students were from junior middle school, representing 69.0%, while 2,669 were from senior high school, making up 31.0%. Additionally, 2,704 students were boarding students, accounting for 31.4%. Various forms of parental absence had a significant impact on grade, household registration, age, boarding at school, and mental health (all *p* values <0.001), as shown in Table 1.

**Table 1:** Demographic characteristics of study participants, n (%).

		NLBC	LBC	DC	DLC	χ^2^/Z	*p*值
Gender	Boys	2,411(28.0)	1,444(16.8)	238(2.8)	447(5.2)	6.430	0.092
	Girls	2,162(25.1)	1,257(14.6)	263(3.1)	384(4.5)		
Grade	Junior high school	3,506(40.7)	1,653(19.2)	361(4.2)	417(4.8)	35.796	<0.001
	Senior high school	1,067(12.4)	1,048(12.2)	140(1.6)	414(4.8)		
Only child	NO	4,022(46.7)	2,437(28.3)	371(4.3)	570(6.6)	3.043	<0.001
	YES	551(6.4)	264(3.1)	130(1.5)	261(3.0)		
Home place	Urban	3,091(35.9)	1,016(11.8)	314(3.6)	239(2.8)	36.515	<0.001
	Rural	1,482(17.2)	1,685(19.6)	187(2.2)	592(6.9)		
Boarding school	NO	3,677(42.7)	1,474(17.1)	383(4.5)	368(4.3)	6.395	<0.001
	YES	896(10.4)	1,227(14.3)	118(1.4)	463(5.4)		
Age		14(12,18)	15(12,18)	15(12,18)	14(12,18)	79.221	<0.001
Depression		4(0,21)	4(0,21)	4(0,21)	2(0,21)	91.764	<0.001
Anxiety		4(0,21)	4(0,21)	4(0,21)	2(0,21)	97.892	<0.001
Stress		6(0,21)	6(0,21)	6(0,21)	4(0,21)	77.924	<0.001

### Mental health status of middle school students

The results of the univariate analysis revealed that senior high school students had significantly lower incidences of depressive symptoms, anxiety symptoms, and stress compared to junior middle school students (*p* < 0.001). Female students exhibited significantly higher levels of anxiety than male students (*p* < 0.001). Only children showed significantly fewer depressive symptoms compared to non-only children (*p* < 0.05). Boarding students had a significantly higher incidence of depressive symptoms, anxiety symptoms, and stress than non-boarding students (*p* < 0.001).

In the DLC group, 6.7% reported experiencing depressive symptoms, including 1.2% with moderate depression, 0.6% with severe depression, and 0.6% with extreme depression. Additionally, 6.1% reported anxiety symptoms and 8.1% reported stress. Parental separation and migration were found to have a significant impact on the severity of depressive symptoms, anxiety symptoms, and stress (all *p* values <0.001). Compared to other groups, middle school students in the DC and DLC groups displayed more severe mental health issues, as indicated in Tables 2, 3.

**Table 2:** Univariate analysis of general information of middle school students n(%).

		Depression(yes)	Anxiety(yes)	Stress(yes)
Grade	Junior high school	1,122(13.0)	1,406(16.3)	665(7.7)
	Senior high school	762(8.9)	899(10.4)	395(4.6)
χ^2^		97.089**	93.634**	22.078**
Gender	Boys	965(11.2)	1,141(13.1)	546(6.3)
	Girls	919(10.7)	1,164(13.5)	514(6.0)
χ^2^		2.273	13.352**	0.751
Only child	NO	1,588(18.5)	1974(22.9)	915(10.6)
	YES	296(3.4)	331(3.8)	145(1.7)
χ^2^		5.591*	0.314	0.113
Boarding school	NO	742(8.6)	906(10.5)	394(4.6)
	YES	1,142(13.3)	1,399(16.3)	666(7.7)
χ^2^		71.004**	88.740**	18.548**
Home place	Urban	906(10.5)	1,088(12.6)	535(6.2)
	Rural	978(11.4)	1,217(14.1)	525(6.1)
χ^2^		35.668**	60.805**	6.582*

*p < 0.05, **p < 0.001.

**Table 3:** Employs the DASS-21 scale and its sub-scales to evaluate the incidence of stress, anxiety, and depression in relation to parental divorce and migration status n(%).

Variable	None	Mild	Moderate	Severe	Very severe	*p*-value
Depression						
NLBC	3,714(43.2)	352(4.1)	355(4.1)	79(0.9)	73(0.8)	<0.001
LBC	2,150(25.0)	206(2.4)	245(2.8)	44(0.5)	56(0.7)	
DC	364(4.2)	49(0.6)	56(0.7)	14(0.2)	18(0.2)	
DLC	567(6.6)	86(1.0)	125(1.5)	26(0.3)	27(0.3)	
Anxiety						
NLBC	3,521(40.9)	478(5.6)	295(3.4)	120(1.4)	159(1.8)	<0.001
LBC	1925(22.4)	327(3.8)	256(3.0)	91(1.1)	102(1.2)	
DC	331(3.8)	75(0.9)	45(0.5)	15(0.2)	35(0.4)	
DLC	524(6.1)	112(1.3)	98(1.1)	36(0.4)	61(0.7)	
Stress						
NLBC	4,068(47.3)	235(2.7)	168(2)	78(0.9)	24(0.3)	<0.001
LBC	2,372(27.6)	154(1.8)	109(1.3)	47(0.5)	19(0.2)	
DC	423(4.9)	34(0.4)	20(0.2)	15(0.2)	9(0.1)	
DLC	683(7.9)	64(0.7)	49(0.6)	23(0.3)	12(0.1)	

### Multifactorial analysis of the factors affecting stress, anxiety, and depression in middle school students

The logistic regression analysis of factors affecting stress, anxiety, and depression revealed several statistically significant predictive factors for mental health problems. Stress, anxiety, and depression were used as dependent variables (1 = YES, 0 = NO), while parental absence (0 = NLBC, 1 = LBC, 2 = DC, 3 = DLC) served as independent variables. Additionally, grade (1 = junior high school, 2 = senior high school), gender (1 = girls, 2 = boys), age, only child (1 = YES, 0 = NO), and boarding at school (1 = YES, 0 = NO) were included as covariates for the regression analysis. The results revealed that various forms of parental absence had a significant impact on depression, anxiety, and stress. Specifically, The combined impact of divorce and left-behind children (DLC) creates a synergistic effect, resulting in psychological risks (OR = 1.623–1.725, all *p* < 0.001), that are significantly higher than LBC or DC. Senior high school students (OR = 1.486, *p* < 0.001) and students living in boarding schools (OR = 1.155, *p* = 0.037) also showed significantly higher levels of depression, anxiety, and stress. Female students (OR = 1.213, *p* < 0.001) exhibited notably higher levels of anxiety compared to their male counterparts, as shown in Table 4. The Hosmer-Lemeshow test confirmed that the model fit was good, *p* > 0.05, indicating a satisfactory fit of the model.

**Table 4:** Multifactor analysis of factors influencing stress, anxiety, and depression in middle school students.

Variable	*B*	*SE*	*Wales* χ^2^	*p*-value	*OR*(95%*CI*)
Depression					
Parental absence			51.057	<0.001	
LBC	0.161	0.062	6.844	0.009	1.175(1.041–1.325)
DC	0.466	0.108	18.729	<0.001	1.594(1.290-1.968)
DLC	0.545	0.087	39.707	<0.001	1.725(1.456–2.044)
Senior high school	0.396	0.067	34.986	<0.001	1.486(1.303–1.695)
Boarding school(YES)	0.144	0.069	4.337	0.037	1.155(1.009–1.322)
Anxiety					
Parental absence			56.901	<0.001	
LBC	0.195	0.057	11.537	0.001	1.215(1.086–1.359)
DC	0.513	0.101	25.630	<0.001	1.670(1.369–2.037)
DLC	0.517	0.083	39.034	<0.001	1.677(1.426–1.972)
Senior high school	0.312	0.063	24.366	<0.001	1.366(1.207–1.547)
girls	0.193	0.049	15.281	<0.001	1.213(1.101–1.336)
Boarding school(YES)	0.224	0.065	11.955	0.001	1.215(1.086–1.359)
Stress					
Parental absence			27.309	<0.001	
LBC	0.067	0.076	0.781	0.377	1.070(0.921–1.243)
DC	0.383	0.132	8.415	0.004	1.467(1.132–1.901)
DLC	0.484	0.104	21.675	<0.001	1.623(1.324–1.991)
Senior high school	0.272	0.070	15.072	<0.001	1.312(1.144–1.505)

## Discussion

This study represents the first comprehensive investigation into the differential impacts of various forms of parental absence on the psychological well-being of middle school students in western China. Against the backdrop of rapid economic development and profound social transformation, parental absence has emerged as a multifaceted phenomenon with diverse manifestations. A significant proportion of Chinese children experience prolonged separation from their parents, primarily due to either rural-to-urban labor migration or family dissolution resulted from divorce. These circumstances necessitate a thorough examination of the psychological adaptation challenges faced by these adolescents.

This study is the first to systematically compare the psychological well-being of middle school students in rural areas of western China based on different types of parental absence. Utilizing the DASS-21, the study reveals that a substantial proportion (at least 10%) of the surveyed adolescents exhibited clinically significant levels of negative emotional states, including stress, anxiety, and depression. The analysis identified both parental divorce and parental migration as significant predictors of compromised mental health outcomes. Notably, DLC emerged as a particularly important risk factor, exerting a disproportionately severe impact on adolescent psychological well-being. These findings underscore the critical need for targeted interventions and support systems to address the unique psychological challenges faced by adolescents experiencing different forms of parental absence in China’s rapidly changing social landscape.

The findings of this study demonstrate that DLC exerts a profound impact on adolescent psychological well-being, particularly manifesting through elevated rates of depressive symptoms (6.7%), anxiety disorders, and chronic stress responses. This dual-stressor phenomenon appears to generate compounded psychological effects, with the interaction between family dissolution and parental migration producing significantly greater mental health risks than either factor alone—particularly evident in depression and anxiety metrics where synergistic effects are most pronounced. Notably, while adolescents from DC experience the trauma of familial fragmentation, they typically retain consistent parental support from the custodial parent. In contrast, the DLC cohort faces a dual deprivation: the structural collapse of nuclear family units combined with physical separation from one or both parents due to labor migration. This unique circumstance creates an emotional support vacuum, leaving adolescents without stable caregiving frameworks during critical developmental stages. The data suggest distinct mechanistic pathways through which parental separation and migration independently undermine mental health, while their co-occurrence establishes multiplicative risk factors. The migration component appears to exacerbate existing vulnerabilities from family dissolution by disrupting established support networks, compromising attachment security, and introducing additional socioeconomic stressors. This convergence of adversities may overwhelm adolescent coping capacities, thereby compounding challenges in emotional regulation, identity formation, and psychosocial adaptation—critical developmental tasks during adolescence. These findings align with existing literature emphasizing the cumulative risk model of childhood adversity (21, 29), while extending current understanding through quantification of interaction effects between family structure changes and migration-related stressors (30). The results underscore the need for targeted interventions to address both familial and socioecological dimensions of psychological distress in this vulnerable population.

From a theoretical perspective, parental divorce typically involving substantial alterations in family structure, intensified familial conflicts, and deteriorated parent–child relationships, all of which can profoundly impact the psychological well-being of middle school students. Empirical evidence indicates that parental disputes and emotional conflicts during the divorce process can subject children to prolonged states of tension and anxiety, rendering them more susceptible to negative emotional states such as depression and diminished self-esteem (31–33). Indeed, adolescents from divorced families demonstrate increased vulnerability to anxiety (5) and depression (34). From a social support framework, parental divorce often disrupts children’s social networks through changes in living arrangements or school transitions, potentially dismantling existing support systems and impeding their ability to access adequate emotional support and social resources in a timely manner (35).

Children from LBC families face the challenge of parental absence, yet their family structure remains relatively intact, enabling them to receive some emotional support and a sense of belonging within a complete family framework. In contrast, the DLC must contend not only with the absence of one or both parents but also with the additional strain of family breakdown. This dual challenge significantly heightens psychological risks compared to LBC from single-parentfamilies. While children from DC families experience the pain of family fragmentation, the presence of at least one parent provides essential care and emotional support. Parental migration presents a dual socioeconomic dynamic: while it can enhance household income and provide children with improved educational prospects, it may simultaneously compromise caregiving capacity due to reduced parental availability (36). This contrasts sharply with the consequences of parental divorce, which frequently triggers economic deterioration and consequent downward mobility in children’s socioeconomic trajectories during formative years (5). In our study sample from northeastern Sichuan, parental migration affected 41.0% of middle school students. Notably, these adolescents reported comparatively lower levels of migration-related stigma, a finding consistent with broader sociological observations (37). However, this apparent social adaptability masks deeper relational vulnerabilities. Empirical evidence indicates that insufficient parental interaction and prolonged separation may erode parent–child relational bonds and serve as risk factors for psychological distress (14). The developmental implications are particularly acute during adolescence, a critical phase for socioemotional skill acquisition and identity formation (38). Of particular concern is the attenuation of parental support mechanisms during this transitional period, given that secure parent–child relationships constitute fundamental protective factors for adolescent mental health (39).

Our study also found that boarding school attendance has an impact on middle school students’ symptoms of depression and anxiety. Compared to day students, those in boarding schools face a higher risk of experiencing anxiety and depression, which aligns with previous research (40). Although attending a boarding school involves separation from parents, this is fundamentally different from the separation caused by parental divorce or migration. In boarding schools, the separation is typically driven by educational or family-specific arrangements, and students often have regular opportunities for parent–child interaction. However, the communal living environment in boarding schools may expose students to negative peer influences, increasing their susceptibility to bad habits, undesirable behaviors, and potential bullying, ultimately affecting both their physical and mental well-being (41). On the other hand, some research suggests that boarding schools can, to some extent, reduce the risk of psychological problems. For instance, the structured communal living environment, with student caregivers assigned by the school, helps ensure the health and safety of boarding students (42). Additionally, psychological counseling from teachers and peer support can help vulnerable students overcome challenges (43).

Gender is another crucial factor influencing mental health. This study found that girls are at a significantly higher risk of anxiety than boys, which aligns with previous research (18, 44). This disparity may be attributed to societal and cultural expectations surrounding emotional expression and gender roles. Girls are often socialized to be more emotionally attuned and expressive, which may lead to a greater internalization of stress and emotional distress, particularly in response to family changes such as divorce or relocation (45, 46). The study also revealed that grade level significantly impacts the mental health of middle school students, with senior high school students exhibiting higher risks of depression, anxiety, and stress. This is consistent with previous research (47, 48), which suggests that the transition to senior high school brings increased academic pressures, social challenges, and uncertainties about the future. This underscores the importance of parental roles and presence in adolescent mental health, as emotional support and companionship from parents play a vital role in supporting the psychological well-being of teenagers (49).

Our study has several limitations that should be acknowledged. First, the cross-sectional nature of the research design precludes the establishment of causal relationships. Future longitudinal studies are warranted to investigate the causal mechanisms and dynamic associations between childhood parental absence and mental health outcomes. Second, the reliance on self-reported data may introduce potential recall bias, which could affect the accuracy of the findings. Third, though the study findings may offer insights into similar contexts, caution should be exercised when generalizing these results to all Chinese adolescents. It is worth noting that Sichuan Province shares demographic similarities with other regions in China, such as Henan, Guizhou, and Guangdong, which also have substantial population of left-behind children. Fourth, the study focus on parental absence as a primary factor, though inclusive of different forms such as divorce and migration, does not allow for a detailed comparison of mental health outcomes and risk behaviors across specific types of absence, particularly between children of single-parent migrants and those with both parents migrating. Furthermore, the investigation was constrained to a limited set of potential determinants, excluding other potentially influential variables such as long-term caregiving arrangements and family social capital. Finally, the exclusive reliance on children’s self-reported data represents another limitation. Future research should adopt a more comprehensive approach to examine family processes, including but not limited to repeated divorces, remarriages, and ongoing family conflicts, and their potential roles in mitigating or exacerbating the psychological impacts of divorce on children.

## Conclusion

Despite the aforementioned limitations, our study underscores the significant mental health risks associated with various forms of parental absence among adolescents in Sichuan region. Both parental separation and migration were found to have a profound impact on adolescent mental health, notably increasing the risks of depression, anxiety, and stress. These findings highlight the critical need for social support systems to prioritize and strengthen the emotional connection between parents and children, particularly in cases of parental separation or migration. By fostering stronger parent–child bonds and providing targeted emotional support, it may be possible to mitigate the adverse psychological effects caused by parental absence and help adolescents better overcome these mental health challenges.

## Data Availability

The raw data supporting the conclusions of this article will be made available by the corresponding authors.

## Footnotes

^1^ https://www.wjx.cn
